# To predict the prognosis of adolescents with anorexia nervosa leaving inpatient treatment: the development and initial evaluation of a novel tool to be used by a multi-disciplinary team

**DOI:** 10.1007/s40519-025-01731-6

**Published:** 2025-03-07

**Authors:** Eleanor Herrmann, Hayley Johns, Emma M. Giles, Philippa McQuilton, Sarah Astbury, Rachel V. Matthews, J. Hubert Lacey

**Affiliations:** 1 Schoen Clinic Newbridge, Birmingham, UK; 2 St George’s, University of London, London, UK

**Keywords:** Eating disorders, Anorexia nervosa, Questionnaire, MDT-rated, Prognosis, Short-term outcome, Adolescents, Evaluation

## Abstract

**Purpose:**

This study aimed to develop and evaluate the Newbridge Prognosis Score (NPS), a prognostic questionnaire designed for use by a multidisciplinary team (MDT) when an anorexia nervosa (AN) patient is discharged from an inpatient treatment facility for eating disorders. The tool integrates the collective judgment of MDT members to predict short-term outcomes in adolescents with AN, focusing on physical, psychological, and behavioural factors and systematically assessing psychopathology, weight, and continued treatment needs. This information is intended to guide individualized community support, enhance post-discharge recovery, and aid in the allocation of limited community-based resources.

**Methods:**

A group of adolescent girls and boys undergoing inpatient treatment for AN participated in the study. In addition, a matched control sub-sample was created. Upon discharge, the multidisciplinary team scored the Newbridge Prognosis Score (NPS), and follow-up data were collected 6 months later from the young person and/or their parents or carers.

**Results:**

The NPS significantly correlates with key recovery items, such as weight maintenance, lower eating disorder psychopathology, subsequent need for readmission and engagement in follow-up. Higher NPS scores predict poorer outcomes, such as more severe psychopathology, lower weight or weight loss and higher readmission risk at follow-up. Psychological factors are strong predictors of post-discharge prognosis, with the suggestion that those sufferers may require enhanced psychological support. The NPS is more effective at predicting long-term readmission risk than short-term outcomes. However, the NPS explained only a proportion of the variance in these outcomes and sensitivity in predicting readmission within the matched control sample was mixed.

**Conclusions:**

The NPS shows promise as a potential tool for predicting short-term outcomes following AN treatment. While promising, further refinement of the tool is needed, prior to being validated for use in clinical practice.

**Level of evidence:**

Level III. Evidence obtained from a well-designed cohort or case-controlled analytic studies.

## Introduction

Anorexia nervosa (AN) is a severe psychiatric disorder characterized by intense fear or phobia of gaining weight and distorted body image, leading to significant medical and psychological complications. Adolescents with AN have a variable prognosis, with relapse rates remaining alarmingly high. Studies have shown that nearly half of adolescents with AN require readmission to inpatient care, and 25% require further hospitalization within the first-year post-discharge [1, 2].

The transition from inpatient care to community settings is a critical period for adolescents with AN. Predicting prognosis during this phase is essential to allocate resources effectively and ensure tailored support. Factors such as family dynamics, comorbidities, motivation, and treatment adherence have been highlighted as significant predictors of outcomes [3, 4]. Despite extensive research, there is a notable absence of tools specifically designed to predict short-term prognosis in adolescents with AN.

The complexity of AN recovery is compounded by its multidimensional nature, involving physical, psychological, and behavioural components. Weight restoration, while a critical goal, is insufficient as a sole marker of recovery, as psychological factors such as cognitive distortions and emotional dysregulation often persist [5]. Furthermore, family support plays a pivotal role in sustaining recovery, particularly during the challenging transition from inpatient care to community settings [6]. This underscores the need for a comprehensive tool capable of synthesizing these diverse factors to predict outcomes more accurately [7].

To address this gap, the Newbridge Prognosis Score (NPS) was developed as a multidisciplinary team (MDT)-rated questionnaire. The NPS consolidates the expertise of diverse professionals involved in a patient’s care, including psychiatrists, psychologists, dietitians, and nurses, to systematically evaluate key prognostic factors. Its development was informed by both empirical research and clinical insights, ensuring that it reflects the complexities of AN recovery [8].

This pilot study aimed to evaluate the ability of the NPS to predict short-term outcomes, including weight restoration, psychopathology, and readmission rates, in adolescents with AN following inpatient treatment. By identifying adolescents at greater risk for poor outcomes, the NPS has the potential to guide resource allocation and enhance care transitions [9].

## Methods

### Development of the NPS

The NPS was conceptualised and developed at Newbridge House, an inpatient unit specializing in adolescent eating disorders. The tool’s development involved an extensive review of the literature on prognostic factors for AN [5, 7]. Following this, focus groups with experienced clinicians were conducted to identify key predictors of short-term outcomes. Iterative revisions were made based on feedback from these focus groups and pilot testing [8].

Pilot testing revealed challenges scoring “psychological change,” leading to its division into “psychological change (thoughts)” and “psychological change (behaviour).” Additional categories were added to account for co-morbidities and family environment, ensuring the NPS comprehensively assessed prognostic factors [6].

The finalised NPS comprises 10 items addressing physical restoration, psychological changes, family dynamics, and community transition (see Table 1). Each item is scored from 1 to 4, with higher scores indicating poorer prognosis. The total score is categorised into five prognosis levels: excellent (10–17), good (18–21), average (22–24), below average (25–28), and poor (29–40) [9].

**Table 1:** Newbridge prognosis score

NPS Item	Version of NPS first present	Description of Item	Additional Scoring Guidance
Physical Restoration	1	Consider weight restoration and maintenance period	
Community Team	1	Consider community team engagement with inpatient team, communication, community package, relationship with young person and family	
Family Burden	2	Consider other physical and mental health problems within the family and the impact this has on the young person. Also consider any additional stress on the family and the impact on family resources and emotional energy	Additional stresses on the family may include, grandparents’ health, financial stress, job insecurity and relationship breakdown
Patient Co-morbidity	2	Consider co-morbid mental illness, Autism Spectrum Disorder, Learning Disability, physical illness and the impact on the young person	Scoring team may also consider relevant co-morbid presentations which are not diagnosed
Mother’s Insight and Support	1	Consider mothers engagement with the care team, and her ability to support the child to make positive changes	If mother is absent or deceased, scoring team to consider the impact of this on the young person (minor, moderate or detrimental to recovery)
Father’s Insight and Support	1	Consider fathers engagement with the care team, and his ability to support the child to make positive changes	If father is absent or deceased, scoring team to consider the impact of this on the young person (minor, moderate or detrimental to recovery)
Psychological Change (Thoughts)	2 (Present as psychological change in version 1, then item divided into change in thoughts and change in behaviour in version 2)	Does the young person have good insight into their difficulties, have they made positive changes related to ED thoughts	Scoring team to consider the use of the Mental Health Act during the admission and whether the young person is being discharged on a Community Treatment Order
Psychological Change (Behaviour)	2 (Present as psychological change in version 1, then item divided into change in thoughts and change in behaviour in version 2)	Does the young person have good insight into their difficulties, have they made positive changes related to ED behaviours	
Transition to Community	1	Consider school reintegration, home leave, hobbies	
Motivation	1	Consider whether the young person has genuine motivation for recovery and is actively engaged in the treatment programme	

This scoring system allows MDTs to summarize and communicate risk factors effectively, ensuring a unified understanding of patient needs at the time of discharge. In addition, specific subscales within the NPS highlight areas of staff concern, such as family readiness for reintegration or ongoing psychological vulnerabilities. This makes the NPS a versatile and detailed tool, the results of which can be transferred to other clinicians.

### Participants

184 patients were discharged between March 2018 and October 2021; 67 of these patients (64 girls and 3 boys) completed the 6-month follow-up questionnaires (36.4%) Participants were aged 10.5–18 years (mean age = 15.9 years). The mean percentage of median BMI (%mBMI) at admission was 76.4%, improving to 97.4% at discharge. A matched control sub-sample of all patients that were readmitted to Newbridge House was created to assess readmission outcomes [1]. These patients (*N* = 15) had been readmitted between 1- and 17-month post discharge and were matched on age of illness onset and age at first admission with patients that had reported no readmission after 6-month post-discharge (*N* = 15). Inclusion criteria included a primary diagnosis of AN based on DSM-5 criteria, while those with severe comorbid psychiatric conditions, such as psychosis or autism spectrum disorder, were excluded to maintain sample homogeneity.

### Measures

- *Eating Disorders Examination Questionnaire (EDE-Q):* This 28-item self-report tool evaluates eating disorder psychopathology, including dietary restraint and shape concerns. A global score of ≥ 4 was used to indicate clinical significance [5].
- *Clinical Information:* Data on weight, height, and readmission status were collected through parent and patient questionnaires [7].
- *Family Support Index (FSI):* A supplementary questionnaire evaluated the family’s readiness to support post-discharge care. Higher scores indicated greater readiness and engagement [8].

### Procedure

During the final MDT meeting before discharge, the NPS was completed collaboratively. Six-month post-discharge, follow-up questionnaires were sent to participants and their families, including the EDE-Q and clinical information forms. Completion was encouraged through reminders and pre-paid return envelopes. Data were anonymized to ensure participant confidentiality. MDT members were trained on scoring protocols to enhance reliability and consistency [9].

### Statistical analysis

Data were analysed using SPSS 28 (IBM). Spearman’s correlations assessed relationships between NPS scores and outcomes. Independent *t* tests compared groups with positive and negative outcomes. Logistic and linear regressions evaluated the predictive power of NPS scores for weight restoration, psychopathology, and readmission [6]. Receiver operating characteristic (ROC) analysis was conducted to evaluate the sensitivity and specificity of NPS scores for predicting readmission. Statistical significance was set at *p* < 0.05 for all tests.

## Results

### NPS scores and response to follow-up

Participants who responded to follow-up questionnaires had significantly lower mean NPS scores (n = 67, *M* = 20.9, SD = 6.04) compared to non-responders (n = 117, *M* = 23.3, SD = 5.76). This finding suggests that individuals with higher NPS scores were less likely to engage in follow-up, indicating a potential association between poorer prognosis and non-compliance. [5] Not completing follow-up questionnaires was more prevalent among participants with higher levels of psychopathology and lower family support scores, emphasizing the importance of these factors in post-discharge recovery [7].

### Correlation with outcomes

#### 1/Eating disorder psychopathology

**Fig. 1:**
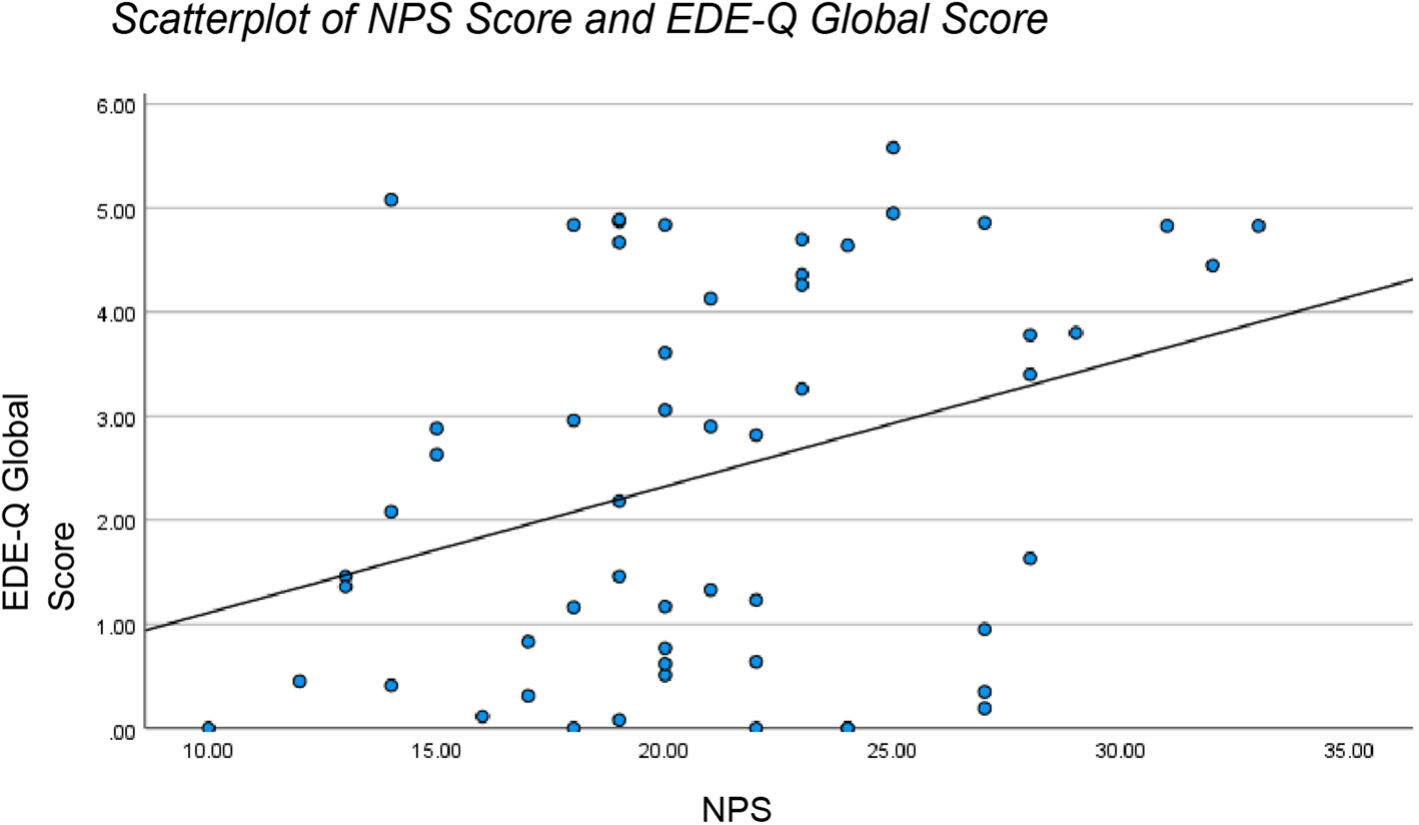
Scatterplot of NPS Score and EDE-Q Global Score. A simple linear regression was used to test if the NPS Score significantly predicted EDE-Q Global Score. The fitted regression model was EDE-Q Score = − 0.11 + 0.12* (NPS Score). The results of the regression indicated that NPS Score explained 11.4% of the variation in EDE-Q Global Score (*F* (1, 54) = 6.93, *p* = .011). These results were significant at the *p* < .05 level

Higher NPS scores were significantly associated with higher EDE-Q global scores (*r* = 0.337, *p* < 0.005), indicating greater eating disorder psychopathology [8]. Subscale analysis revealed that psychological change items within the NPS were the strongest predictors of higher EDE-Q scores, perhaps highlighting the need for targeted psychotherapy [9] (Fig. 1).

#### 2/Percentage healthy weight

**Fig. 2:**
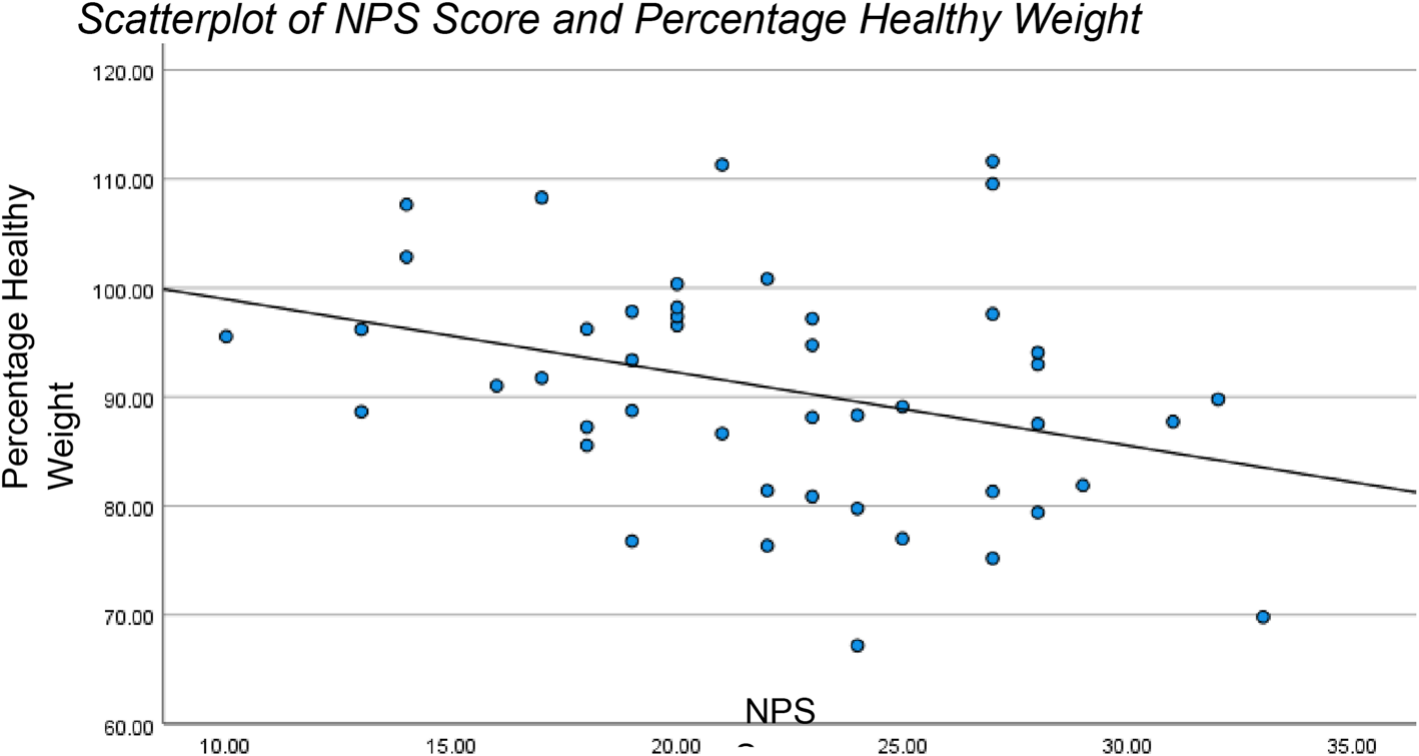
Scatterplot of NPS Score and percentage healthy weight

NPS scores were negatively correlated with weight maintenance (%mBMI) at 6-month post-discharge (*r* = − 0.339, *p* = 0.011). Adolescents with lower NPS scores were more likely to maintain healthy weight, though a simple linear regression showed that the NPI scores explained only 11.3% of the variance in %mBMI. Notably, weight outcomes were more favourable among participants with high family support scores [10] (Fig. 2).

#### 3/Readmission rates

Higher NPS scores predicted an increased likelihood of readmission within 6 months. ROC analysis demonstrated moderate sensitivity (72%) and specificity (68%) for predicting readmission within 6 months [6].

A logistic regression analysis was performed with readmission at 6 months as the dependent variable, and NPS score as the predictor variable. A total of 66 cases were analysed and the full model significantly predicted readmission status at 6 months [omnibus chi-square = 5.895, df = 1 and *p* = 0.015 (< 0.05)]. The model accounted for between 8.5% and 14.9% of the variance in readmission, with 100% of people not readmitted at 6 months correctly predicted. However, only 10% of predictions for the readmitted at 6-month group were accurate. In the overall regression model of readmission and non-readmission, 86.4% of predictions were accurate.

### Matched control analysis

In the matched control group, adolescents readmitted within 17 months had significantly higher NPS scores (*M* = 22.3, SD = 4.30) compared to those who were not readmitted (*M* = 18.1, SD = 5.18). Logistic regression demonstrated that NPS scores predicted 22.5% of the variance in readmission outcomes. Further analysis revealed that participants with poor prognosis scores (> 28) had a 2.5-fold increased risk of readmission compared to those with excellent or good scores.

Any reader requiring further information regarding the results or other data in this study should contact the corresponding author at hlacey@sgul.ac.uk.

## Discussion

The results of this study underscore the promise of the Newbridge Prognosis Score (NPS) as a predictive tool for adolescents with anorexia nervosa (AN) transitioning from inpatient treatment to community settings. Significant correlations between NPS scores and critical recovery outcomes, such as weight restoration, eating disorder psychopathology and subsequent readmission, highlight the tool’s utility in identifying adolescents at greater risk of poor outcomes. Nonetheless, the modest variance explained by NPS scores in predicting outcomes such as readmission underlines the complexity of recovery in AN.

In the matched control group, patients who subsequently required readmission had significantly higher NPS scores compared to those who were not readmitted. Participants with poor prognosis scores had a 2.5-fold increased risk of readmission compared to those with excellent or good scores. These findings suggest that the NPS provides valuable insights into long-term risks and can inform ongoing care planning.

One key observation is that NPS scores appear to capture factors beyond traditional metrics like weight restoration. The inclusion of psychological and family dynamics aligns with research emphasizing the role of holistic recovery in AN [6]. Furthermore, the tool’s ability to predict compliance with follow-up highlights its potential value for structuring post-discharge care plans.

Psychological change items within the NPS were the strongest predictors of higher EDE-Q scores, perhaps highlighting the need for targeted psychotherapy [9]. Furthermore, individuals with higher NPS scores were less likely to engage in follow-up, indicating a potential association between poorer prognosis and non-compliance.

However, the NPS’s limitations also warrant attention. While significant, the explained variance in outcomes remains limited, suggesting that other critical variables, such as the therapeutic alliance and social support systems, may not be adequately captured. In addition, the tool’s sensitivity in predicting short-term readmissions—a crucial clinical metric—remains modest. Expanding the scoring system or incorporating additional parameters could enhance its predictive accuracy.

### Strengths and limitations

The strengths of the NPS lie in its design and practical applicability. Developed through iterative feedback from experienced clinicians, the tool benefits from excellent inter-rater reliability and ease of integration into MDT workflows. By providing a structured framework, the NPS facilitates systematic assessments and improves communication within multidisciplinary teams.

However, the study’s limitations must be acknowledged. Attrition bias, evident in lower response rates among participants with poorer outcomes, may skew results. In addition, the study’s relatively small sample size and single-centre design limit the generalizability of findings. The exclusion of variables such as treatment duration and family functioning further constrain the tool’s explanatory power.

### Clinical implications

From a clinical perspective, the NPS offers a promising approach to structuring care transitions for adolescents with AN. By identifying individuals at high risk for relapse, the tool allows MDTs to prioritise resources and design targeted interventions. For instance, adolescents with higher NPS scores could benefit from intensified outpatient support, including family-based therapy or regular weight monitoring. Furthermore, the tool’s integration into discharge planning could improve coordination between inpatient and community services, addressing a critical gap in current care models.

The study also underscores the importance of holistic assessments in AN recovery. The inclusion of psychological and behavioural factors in the NPS aligns with contemporary treatment paradigms that emphasize the interplay between mental and physical health. Future iterations of the tool could further refine this approach by incorporating validated measures of therapeutic alliance and social support.

### Future directions

Future iterations of the NPS should expand the scoring scale to allow for greater precision in assessments, enabling the tool to capture subtle variations in patient prognosis more effectively. Additional prognostic factors, such as comorbid conditions and family functioning, should also be incorporated to enhance the tool’s comprehensiveness and accuracy. Moreover, follow-up periods should be extended to evaluate long-term outcomes and validate the NPS’s utility over time, as this would provide a clearer understanding of its predictive power. Larger, multicentre studies are needed to improve the generalizability of findings and to establish normative data for the NPS, ensuring it can be effectively applied in diverse clinical settings.

### What is already known on this subject

At present there is no standardised, reliable and valid method of collating the clinical opinions of an MDT at the point an anorexia nervosa patient is discharged from hospital to the community-based services. Such a questionnaire could guide individualized community support, enhance post-discharge recovery, and aid in the allocation of limited community-based resources.

### What this study adds

This study demonstrates it is possible to develop a prognostic questionnaire which integrates the collective judgment of MDT members to predict short-term outcomes in adolescents with AN. The questionnaire focusing on physical, psychological, and behavioural factors and systematically assesses psychopathology, weight, and continued treatment needs. The NPS significantly correlates with key recovery items, such as weight maintenance, lower eating disorder psychopathology, subsequent need for readmission and engagement in follow-up. Further refinement is needed.

## Conclusion

The Newbridge Prognosis Score represents a promising step toward standardised short-term outcome prediction in adolescents with AN. While preliminary findings support its clinical utility, further refinement and validation are necessary before widespread implementation. By addressing current limitations, the NPS has the potential to become a valuable tool in the management of adolescent AN.

## Data Availability

No data sets were generated or analysed during the current study.
